# Accelerated Increase in *Candida auris* Bloodstream Infections during COVID-19 Pandemic, South Africa 

**DOI:** 10.3201/eid3204.251407

**Published:** 2026-04

**Authors:** Husna Ismail, Olga Perovic, Ruth Mpembe, Warren Lowman, Chetna Govind, Pieter Ekermans, Waasila Jassat, Richard Welch, Nelesh P. Govender

**Affiliations:** National Institute for Communicable Diseases, a Division of the National Health Laboratory Service, Johannesburg, South Africa (H. Ismail, O. Perovic, R. Mpembe, W. Jassat, R. Welch, N.P. Govender); University of the Witwatersrand, Johannesburg South Africa (R. Mpembe, W. Lowman, N.P. Govender); Vermaak and Partners/Pathcare Pathologists, Pretoria, South Africa (W. Lowman); Wits Donald Gordon Medical Centre, Johannesburg (W. Lowman); Lancet Laboratories, Durban, South Africa (C. Govind); Ampath Microbiology National Reference Laboratory, Centurion, South Africa (P. Ekermans)

**Keywords:** *Candida auris*, fungi, fungemia, COVID-19, coronavirus disease, SARS-CoV-2, severe acute respiratory syndrome coronavirus 2, viruses, respiratory infections, zoonoses, interrupted time series, South Africa

## Abstract

The COVID-19 pandemic coincided with rising secondary bloodstream infections (BSIs) from multidrug-resistant organisms, including *Candida* *auris*. To assess candidemia trends, we conducted a retrospective analysis of blood culture isolates from public and private laboratories in South Africa taken during January 2019–June 2022. We evaluated weekly aggregated *Candida* BSI counts and COVID-19 cases using segmented regression within an interrupted time-series framework. In total, 15,393 candidemia cases were identified, 70% from the private sector. *C. parapsilosis* accounted for 39% of cases, whereas *C. auris* represented 26%. The proportion of *C. auris* increased significantly from 17% in 2019 to 31% in 2021 (p<0.01). After the pandemic onset, *Candida* BSIs rose by 11 cases per week (p = 0.03), largely driven by *C. auris* (+5 cases/week; p<0.01); peaks coincided with COVID-19 waves. Those results highlight an accelerated shift toward *C. auris* in *Candida* BSIs and the urgent need for enhanced surveillance, diagnostics, and infection prevention.

The COVID-19 pandemic, caused by the novel respiratory virus SARS-CoV-2, created strains on the global healthcare system that were experienced mainly in the delivery of acute hospital services (*1*,*2*). Those challenges led to secondary healthcare-associated bloodstream infections (BSIs) caused by multidrug-resistant bacteria and fungi, partially related to increased background use of antimicrobial agents, steroids, and immunomodulatory agents (*3*–*5*). In addition, viral-induced immune-mediated damage by SARS-CoV-2 might have encouraged bacterial and fungal colonization (*6*).

*Candida* BSI is a growing concern worldwide. More than 90% of *Candida* BSIs are caused by 1 of 6 species: *C. albicans*, *Nakaseomyces glabratus* (previously *C. glabrata*), *C. tropicalis*, *C. parapsilosis*, *Pichia kudriavzevii* (previously *C. krusei*), and, increasingly, *C.* *auris (**7**)*. Those 6 species are also included in the World Health Organization Fungal Priority Pathogen List (*8*). In the EUROBACT-2 international cohort study, fungal pathogens accounted for ≈8% (230/2,927) of all healthcare-associated BSIs from 2,600 enrolled patients in 333 intensive care units in 52 countries during June 2019–January 2020 (*8*). *Candida* species were the most common fungi; 58% (133/230) were identified as non–*C.* *albicans* *Candida* species (*9*). *Candida* spp. was the third most common healthcare-associated BSI pathogen (≈12% [333/2,828]) in a prepandemic multicenter study from India (*10*).

The emergence of *C. auris* in South Africa was first recognized retrospectively from a BSI isolate collected in 2009, which was initially misidentified as *C. haemulonii* because of limitations in standard diagnostic methods (*11*). National surveillance during October 2012–November 2016 identified a total of 1,692 cases of confirmed or probable *C. auris* infections across South Africa; cases were concentrated in Gauteng Province, where 92% of cases were reported from private-sector hospitals (*11*). Nationally, the number of cases rose exponentially from 18 in the baseline period (October 2012–November 2013) to 861 in the corresponding final period (October 2015–November 2016) (*11*). By 2016–2017, *C. auris* was the third most common cause of BSI nationally, surpassing most other non–*C. albicans* species (*12*). Molecular epidemiology revealed that isolates in South Africa predominantly belonged to the African clade III, which is genetically distinct and almost universally resistant to fluconazole; ≈6% had reduced susceptibility to amphotericin B (MIC >2 µg/mL) (13). Of note, 5% of isolates were resistant to both fluconazole and amphotericin B (*13*).

The COVID-19 pandemic coincided with a marked increase in *C. auris* outbreaks worldwide, particularly in intensive care settings (*14*). Several contributing factors were identified, including prolonged mechanical ventilation, widespread antimicrobial use, increased indwelling device exposure, and compromised infection prevention protocols (*14*). A systematic review (*14*) documented *C. auris* outbreaks in >10 countries, including India, Brazil, Mexico, Pakistan, the United States, and multiple countries in Europe; reported crude mortality rates among COVID-19 co-infected patients ranged from 30% to 72%. Evidence from Orange County, California, USA, further illustrated this trend (*15*); in long-term acute-care hospitals, the probability of new *C. auris* colonization during the first COVID-19 wave reached 22.5% (95% CI 18.5–26.6) within 30 days of admission. That increase coincided with widespread disruptions in infection prevention practices and shortages of protective equipment, and although incidence declined in later phases of the pandemic, the overall prevalence of colonization remained high (*15*). Schaefer et al. (*16*) reported that *C. auris* incidence at a major academic hospital in New York increased nearly 3-fold, from 2.6 cases/10,000 admissions in 2019 to 7.8 cases/10,000 admissions in 2022 (*16*). Similarly, national surveillance data from China revealed 1,846 laboratory-confirmed *C. auris* cases across 22 provinces; ≈80% of isolates were resistant to fluconazole and ≈7% were resistant to amphotericin B (*17*). 

In this study, we aimed to evaluate the distribution of *Candida* species isolated from blood cultures from both private and public health sectors in South Africa and to describe changes during the COVID-19 pandemic. Permission to conduct this study was obtained from the South African Society of Clinical Microbiology and NHLS, and ethics approval was obtained from the Human Research Ethics Committee (Medical) of the University of the Witwatersrand (clearance no. M210752).

## Materials and Methods

### Study Design, Population, and Setting

We conducted a secondary analysis of blood culture data archived in pathology laboratory information systems from January 1, 2019, through June 30, 2022, in South Africa. The study population consisted of all patients who had a blood culture submitted either to the National Health Laboratory Service (NHLS) or to 1 of 3 large amalgamated private pathology practices (Ampath [https://www.ampath.co.za], Lancet Laboratories [https://www.lancet.co.za], or PathCare/Vermaak and Partners [https://www.vpath.co.za]). The NHLS provides routine diagnostic pathology services to the public health sector, serving ≈83% of the population of South Africa, and has 60 laboratories that offer microbiology testing, including automated blood culture systems and culture identification (*11*). The 3 large private pathology groups provide a full range of microbiology testing for almost all inpatients in South Africa with private health insurance (*11*). The NHLS used several platforms to identify *Candida* to species level at the time of this study: Vitek 2 YST, API 20C Aux, or API ID 32C (bioMérieux, https://www.biomerieux.com); Auxacolor (Bio-Rad Laboratories, https://www.bio-rad.com); and Microscan (Beckman Coulter, https://www.beckmancoulter.com) (*11*). The private pathology groups identified *Candida* primarily using matrix-assisted laser desorption/ionization time-of-flight mass spectrometry methods (Bruker, https://www.bruker.com; or bioMérieux). The total number of blood culture specimens collected during the study period was not available as a denominator. The private health sector represents a small proportion of the general population, and laboratory data from this sector might not be directly comparable with the public sector owing to factors such as specimen-taking practices (*18*). We included patients with blood cultures positive with any of the 6 most common *Candida* species (*C. albicans*, *C. auris*, *N. glabratus*, *P. kudriavzevii*, *C. parapsilosis*, and *C. tropicalis*) in this analysis. Blood culture data from NHLS were obtained from the surveillance data warehouse at the National Institute for Communicable Diseases (NICD) (*19*). Blood culture data from the private sector were obtained through the South African Society of Clinical Microbiology, which has formed a public–private partnership with the NICD (*19*).

### Definitions

We defined a case of *Candida* BSI was defined as illness in a person seen at a healthcare facility in South Africa who had a blood culture from which *C. albicans*, *C. auris*, *N. glabratus*, *P. kudriavzevii*, *C. parapsilosis*, or *C. tropicalis* was isolated. We regarded positive blood cultures with the same organism that had been collected within 30 days of a first positive blood culture as duplicates and excluded them.

### DATCOV and SARS-CoV-2

We extracted COVID-19 hospital admission data from March 5, 2020, through June 30, 2022, from the inpatient national surveillance system, DATCOV. Established in April 2020 by the NICD, DATCOV served as an active surveillance program for COVID-19 hospital admissions for both public and private hospitals in South Africa (*1*). We extracted routine surveillance data for SARS-CoV-2 infections from the reengineered Notifiable Medical Conditions Surveillance System for the period March 1, 2020, through June 30, 2022. In July 2020, the NICD established the Notifiable Medical Conditions Surveillance System, which contains data for all SARS-CoV-2 tests (PCR or antigen detection) conducted in South Africa (*20*). January 2019–February 2020 was considered the prepandemic baseline, and March 2020–June 2022 was the COVID-19 pandemic period.

### Data Management and Analysis

We reported categorical variables for blood culture data in tables as frequencies and percentages or presented them as bar charts and tables. We used the Pearson χ^2^ test or Fisher exact test to compare *Candida* species distribution among years, sectors, and provinces. We performed all analyses using Stata version 15 (StataCorp LLC, https://www.stata.com) or R Studio version 4.5.1 (https://rstudio.com/products/rstudio). We prepared and tabulated blood culture, DATCOV, and SARS-CoV-2 datasets in Microsoft Excel (https://www.microsoft.com). We used those tabulated datasets to plot graphs and apply 30-day moving averages for cases of *C. auris* and *C. parapsilosis* BSI. We conducted interrupted time series analysis to assess the effects of the COVID-19 pandemic on the incidence of *Candida* BSI, with a specific focus on *C. auris*. We defined the intervention point as the onset of the COVID-19 pandemic in South Africa (i.e., March 2020). We aggregated *Candida* BSI and COVID-19 case counts by week and scaled COVID-19 count data to enable visual comparison. We then plotted weekly counts to examine trends before and after the interruption. We used segmented regression to estimate changes in the level and slope of *Candida* BSI incidence associated with the pandemic. We annotated national lockdown levels 1–5 in increasing order of restrictiveness. We used interrupted time series models to evaluate both immediate and gradual changes in weekly *Candida* BSI rates.

## Results

### Distribution of Cases of *Candida* BSI

**Figure 1:**
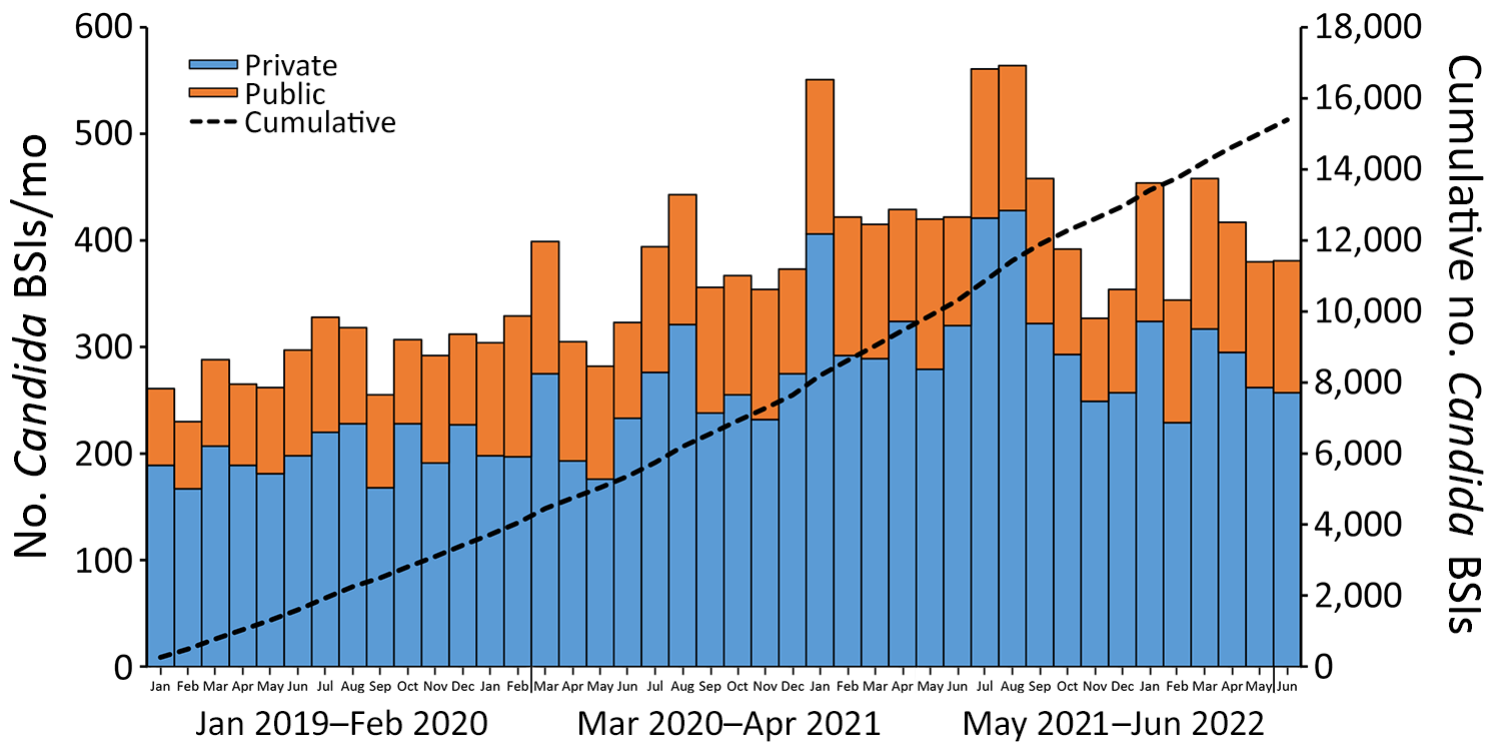
Total number of cases of *Candida *BSI by health sector in study of accelerated increase in *C. auris* BSIs during COVID-19 pandemic, by month and cumulative, South Africa, January 2019–June 2022. Blue bars represent cases in the private sector (n = 10,826), orange bars represent cases in the public sector (n = 4,567), and black dashed line represents the cumulative number of *Candida* BSI cases in South Africa. Scales for the y-axes differ substantially to underscore patterns but do not permit direct comparisons. BSI, bloodstream infection.

Over the 42-month period, 15,393 cases of *Candida* BSI were reported: 3,415 cases in 2019, 4,229 cases in 2020, 5,315 cases in 2021 and 2,434 cases during January–June 2022. The private sector accounted for 70% (10,826/15,393) of all cases. Cases of *Candida* BSI increased across the country, from 4,048 during the 14-month baseline period (January 2019–February 2020) to 5,413 in the first 14 months of the pandemic (March 2020–April 2021), a 34% rise; cases then increased to 5,932 in the following 14-month period (May 2021–June 2022), a further 10% increase (Figure 1). Cases in Gauteng Province accounted for 50% (7,706/15,393) of all *Candida* BSI cases, followed by KwaZulu-Natal Province (14% [2,163/15,393]) and the Western Cape Province (12% [1,809/15,393]). Similar distribution patterns were observed for those provinces in both private and public sectors (Appendix Table).

*C. parapsilosis* accounted for 39% (6,080/15,393) of all *Candida* BSI cases in South Africa, followed by *C. auris* (26% [3,928/15,393]). Nationally, there were notable differences in the distribution of *Candida* species. The relative frequencies of cases of *C. auris* BSI increased from 17% (597/3,415) in 2019 to 31% (1,626/5,315) in 2021 (p<0.01); a corresponding decrease was noted in the percentage of *C. parapsilosis*, from 46% (1,555/3,415) in 2019 to 37% (1,955/5,315) in 2021 (p<0.01), as well as a percentage decrease for *N. glabratus* from 10% (332/3,415) in 2019 to 7% (359/5,315) in 2021 (p<0.01). The relative frequencies of *C. albicans, P. kudriavzevii*, and *C. tropicalis* were relatively unchanged (Table). *C. parapsilosis* was the most common species in 8 of the 9 provinces in South Africa. *C. auris* was the dominant species in the North West Province and accounted for 42% (312/749) of cases (Appendix Figure). In the private sector, *C. parapsilosis* was the dominant species, accounting for 44% (4,742/10,826) of cases, followed by *C. auris,* which accounted for 32% (3,507/10,826) of cases. We observed a more marked increase in the percentage of cases of *C. auris* BSI in the private sector, from 24% (580/2,393) in 2019 to 37% (1,439/3,880) in 2021 (p<0.01); we observed a corresponding decrease in the percentage of *C. parapsilosis*, from 51% (1,217/2,393) in 2019 to 40% (1,567/3,880) in 2021, with a further decrease to 37% (629/1,684) in 2022 (p<0.01). In the public sector, *C. albicans* was the dominant species, accounting for 41% (1,883/4,567) of cases, followed by *C. parapsilosis*, which accounted for 29% (1,338/4,567) of cases. Similar to the private sector, the percentage of cases of *C. auris* BSI in the public sector increased substantially, from 2% (17/1,022) in 2019 to 13% (187/1,435) in 2021 (p<0.01); we noted a corresponding decrease in the percentage of *C. parapsilosis*, from 33% (338/1,022) in 2019 to 27% (388/1,435) in 2021 (p<0.01), as well as a decrease in *N. glabratus* BSIs, from 16% (160/1,022) in 2019 to 12% (169/1,435) in 2021 (p = 0.01) (Table).

**Table:** Distribution of 6 *Candida* species isolated from bloodstream infection by health sector in study of accelerated increase in *Candida auris* bloodstream infections during COVID-19 pandemic, South Africa, January 2019–June 2022

Category	*Candida* species	No. (%)					p value
		2019	2020	2021	2022	Total	
National	*Candida albicans*	793 (23)	959 (23)	1,200 (23)	597 (25)	3,549 (23)	0.26
	*Candida auris*	597 (17)	1,012 (24)	1,626 (31)	693 (28)	3,928 (26)	<0.01
	Nakaseomyces glabratus*	332 (10)	339 (8)	359 (7)	209 (9)	1,239 (8)	<0.01
	*Pichia kudriavzevii*†	63 (2)	93 (2)	73 (1)	31 (1)	260 (2)	0.01
	*Candida parapsilosis*	1,555 (46)	1,734 (41)	1,955 (37)	836 (34)	6,080 (39)	<0.01
	*Candida tropicalis*	75 (2)	92 (2)	102 (2)	68 (3)	337 (2)	0.11
	Total	3,415 (100)	4,229 (100)	5,315 (100)	2,434 (100)	15,393 (100)	
Private	*C. albicans*	364 (15)	431 (15)	605 (16)	266 (16)	1,666 (15)	0.88
	*C. auris*	580 (24)	863 (30)	1,439 (37)	625 (37)	3,507 (32)	<0.01
	*N. glabratus*	172 (7)	163 (6)	190 (5)	127 (8)	652 (6)	0.01
	*P. kudriavzevii*	22 (1)	51 (2)	39 (1)	6 (0.4)	118 (1)	0.01
	*C. parapsilosis*	1,217 (51)	1,329 (46)	1,567 (40)	629 (37)	4,742 (44)	<0.01
	*C. tropicalis*	38 (2)	32 (1)	40 (1)	31 (2)	141 (1)	0.04
	Total	2,393 (100)	2,869 (100)	3,880 (100)	1,684 (100)	10,826 (100)	
Public	*C. albicans*	429 (42)	528 (39)	595 (41)	331 (44)	1,883 (41)	0.11
	*C. auris*	17 (2)	149 (11)	187 (13)	68 (9)	421 (9)	<0.01
	*N. glabratus*	160 (16)	176 (13)	169 (12)	82 (11)	587 (13)	0.01
	*P. kudriavzevii*	41 (4)	42 (3)	34 (2)	25 (3)	142 (3)	0.14
	*C. parapsilosis*	338 (33)	405 (30)	388 (27)	207 (28)	1,338 (29)	0.01
	*C. tropicalis*	37 (4)	60 (4)	62 (4)	37 (5)	196 (4)	0.59
	Total	1,022 (100)	1,360 (100)	1,435 (100)	750 (100)	4,567 (100)	

*Previously *Candida glabrata.*†Previously *Candida krusei.*

**Figure 2:**
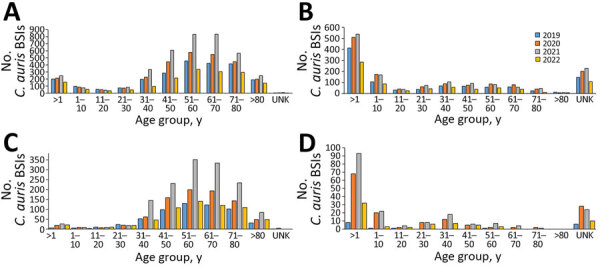
Cases of BSI by type in study of accelerated increase in *Candida auris* BSIs during COVID-19 pandemic, by year and age group, South Africa, January 2019–June 2022. A, B) Total number of cases of *Candida* BSI in private sector (A; n = 10,826) and public sector (B; n = 4,567) (N = 15,393). C, D) Total number of cases of *C. auris* BSI in private sector (C; n = 3,507) and public sector (D; n = 421). BSI, bloodstream infection; UNK, unknown.

Of the 15,393 total cases, 54% (8,333) of patients were male and 46% (7,060) female. The highest number of cases of *Candida* BSI were seen in persons <1 year of age (17% [2,561]), 51–60 years of age (16% [2,480]) and 61–70 years of age (15% [2,348]). When stratified by health sector, 38% (1,745/4,567) of cases in the public sector were in persons <1 year of age, whereas 40% (4,316/10,826) of cases from the private sector were in persons 51–70 years of age (Figure 2, panels A, B). With the exception of 2 age categories (21–30 years of age and 51–60 years of age) in the private sector, we observed distinct differences among the other age categories across all 4 years. We observed that 74% (201/273) of persons <1 year of age with a *C. auris* BSI were from the public sector. In contrast, almost all *C. auris* BSI cases in persons 51–60 years of age (98% [821/834]) and in persons 61–70 years of age (99% [769/775]) were reported from the private sector (Figure 2, panels C, D).

### Distribution of Cases of *Candida* BSI before and during the COVID-19 Pandemic

The first laboratory-confirmed case of SARS-CoV-2 infection was documented in South Africa on March 5, 2020. During the study period, 3,995,881 cases of COVID-19 were reported; 486,789 of those resulted in hospital admission. More than half of COVID-19 hospital admissions (52% [254,975/486,789]) occurred in the public sector.

**Figure 3:**
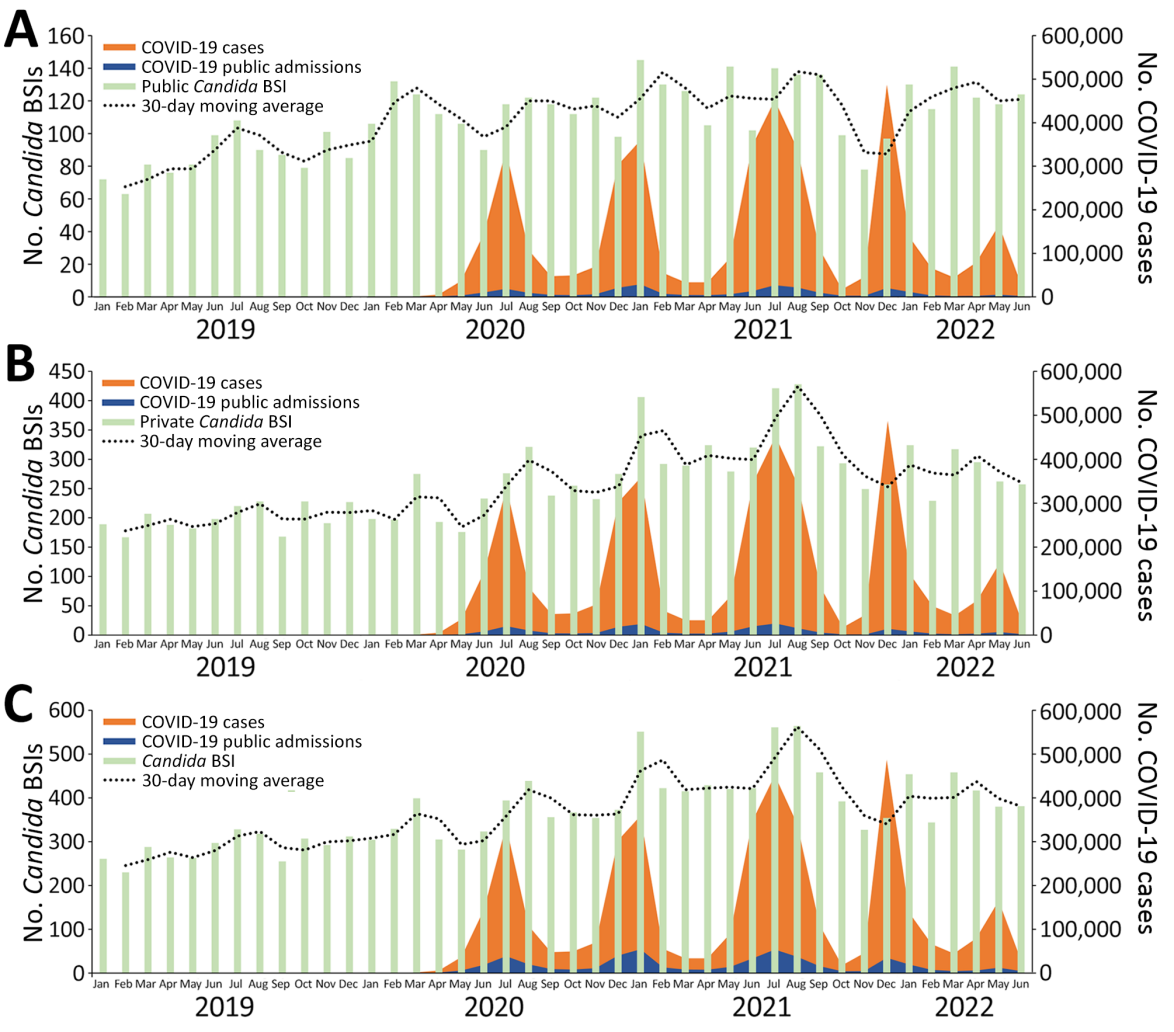
Total number of cases of *Candida* BSIs (n = 15,393), COVID-19 cases (n = 3,995,881), and COVID-19 hospital admissions (n = 486,789), by month and 30-day moving average, in study of accelerated increase in *C. auris* BSIs during COVID-19 pandemic, South Africa, January 2019–June 2022. Numbers are shown for public health sector (A), private health sector (B), and total national (C). Light green bars represent cases of *Candida* BSI (private, n = 10,826; public, n = 4,567), orange bars represent COVID-19 cases, blue bars represent COVID-19 hospital admissions (private, n = 231,814; public, n = 254,975), and black dotted line represents the 30-day moving average number cases. Scales for the y-axes differ substantially to underscore patterns but do not permit direct comparisons. BSI, bloodstream infection.

During the pandemic period (March 2020–June 2022), the number of COVID-19 cases and admissions increased together; the admission peak lagged behind the case peak for each major COVID-19 wave. We observed a temporally related increase in the 30-day moving average of *Candida* BSI cases after each of the 4 waves caused by the ancestral SARS-CoV-2 and 3 variants, Beta, Delta, and Omicron BA.1, in South Africa during the study period. The monthly average number of *Candida* BSI cases was 419 in August 2020, 487 in February 2021, 563 in August 2021, and 438 in April 2022. We also observed fluctuations of the 30-day moving average in *Candida* BSI in the prepandemic period. In the public sector, an increase in the *Candida* BSI 30-day moving average was only noted after the second COVID-19 wave, caused by the Beta variant; a monthly average of 138 cases was reported in February 2021 (Figure 3).

**Figure 4:**
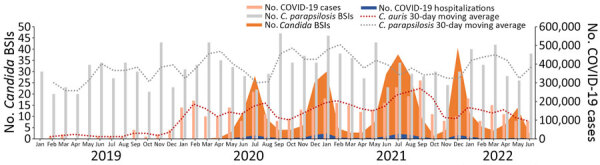
Numbers of *Candida auris* and *C. parapsilosis* BSIs, COVID-19 cases, and COVID-19 hospital admissions, by month, in study of accelerated increase in *C. auris* BSIs during COVID-19 pandemic, South Africa, January 2019–June 2022. Light red bars represent cases of *C. auris* BSI (private n = 3,507; public n = 421), light gray bars represent cases of *C. parapsilosis* BSI (private n = 4,742; public n = 1,338), orange shading represents total number of COVID-19 cases (N = 3,995,881), blue shading represents number of COVID-19 hospital admissions (private n = 231,814; public n = 254,975), dark red dotted line represents 30-day moving average for *C. auris*, and dark gray dotted line represents 30-day moving average for *C. parapsilosis*. BSI, bloodstream infection.

Using *C. parapsilosis* (the most common species in the private sector and second most common species in the public sector) as a comparator, we found that, as the COVID-19 pandemic progressed from March 2020 to June 2022, the average number of cases of *C. auris* BSI increased to the point where *C. parapsilosis* and *C. auris* had similar 30-day moving averages. That phenomenon occurred in both the private and public health sectors, although in the private sector, by the end of the study period, *C. auris* increased from an average of 2 cases/day in February 2019 to 3 cases/day in June 2022 (Figure 4).

### Interrupted Time Series Analysis

**Figure 5:**
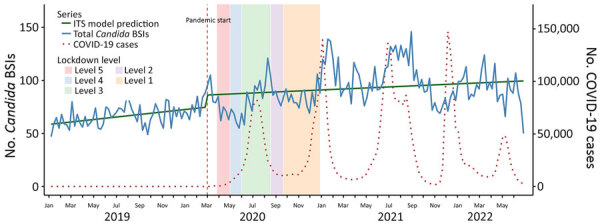
Effects of the COVID-19 pandemic on weekly *Candida* BSI counts in study of accelerated increase in *C. auris* BSIs during COVID-19 pandemic, South Africa, January 2019–June 2022. Vertical dashed red line marks the onset of the COVID-19 pandemic in South Africa (March 5, 2020). Solid green line represents the ITS model prediction for *Candida* BSIs; solid blue line indicates the actual number of *Candida* BSIs. Weekly COVID-19 case counts are scaled (dotted red line) and overlaid for comparison. Background shading indicates timing and duration of national lockdown alert levels: level 5, March 27–April 30, 2020; level 4, May 1–May 31, 2020; level 3, June 1–August 17, 2020; level 2, August 18–September 20, 2020; level 1, September 21–December 28, 2020. BSI, bloodstream infection; ITS, interrupted time series.

**Figure 6:**
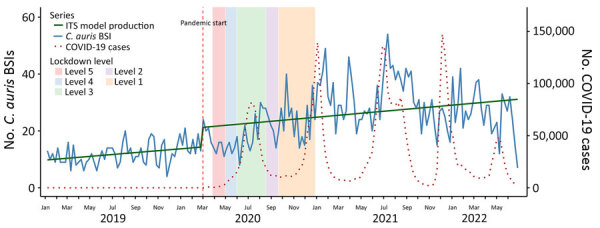
Effects of the COVID-19 pandemic on weekly *Candida auris* BSI counts in study of accelerated increase in *C. auris* BSIs during COVID-19 pandemic, South Africa, January 2019–June 2022. Vertical dashed red line marks the onset of the COVID-19 pandemic in South Africa (March 5, 2020). Solid green line represents ITS model prediction for *C. auris* BSIs; solid blue line indicates the actual number of *C. auris* BSIs. Weekly COVID-19 case counts are scaled (dotted red line) and overlaid for comparison. Background shading indicates timing and duration of national lockdown alert levels: level 5, March 27–April 30, 2020; level 4, May 1–May 31, 2020; level 3, June 1–August 17, 2020; level 2, August 18–September 20, 2020; level 1, September 21–December 28, 2020. BSI, bloodstream infection; ITS, interrupted time series.

Before the COVID-19 pandemic (January 2019–February 2020), the weekly trend in *Candida* BSI was gradually increasing, and the slope before the pandemic was significantly positive (cases increasing by 0.038/week before March 2020; p = 0.02). The onset of the pandemic in March 2020 was associated with a significant increase in *Candida* BSI cases (immediate jump of 11 cases/week; p = 0.03). However, the postintervention slope for *Candida* BSIs did not differ significantly from the prepandemic slope (cases decreasing by 0.023/week; p = 0.20). Peaks in *Candida* BSI were temporally aligned with each of the 4 major COVID-19 waves in South Africa (Figure 5). *C. auris* alone showed a different pattern. Before the COVID-19 pandemic, cases of *C. auris* were increasing, but not significantly (cases increasing by 0.010/week before March 2020; p = 0.37). After the pandemic began, *C. auris* exhibited a sharp immediate increase (*C. auris* cases jumped by 5/week; p<0.01) and continued to increase after March 2020 (cases increasing by 0.032/week; p<0.01) (Figure 6).

## Discussion

After the pandemic, *C. auris* cases of candidemia immediately increased sharply and continued to increase. One quarter of all cases of BSI in South Africa were caused by *C. auris*; more cases of *C. auris* infection in infants were diagnosed in the public sector, and more cases in older age groups were diagnosed in the private sector.

We observed a substantial increase in cases of *Candida* BSI after the peak of COVID-19 hospital admissions. Overall, this trend was similar across all 4 COVID-19 waves and most pronounced for the Delta wave in the Southern Hemisphere in winter of 2021. Fungal infections are a substantial concern in managing patients infected with SARS-CoV-2 (*21*). Our study showed that the number of cases of *C. auris* increased and were similar in monthly averages to cases of *C. parapsilosis*, particularly in the private sector. *C. parapsilosis* and *C. auris* are both species known to cause invasive infections because of their ability to contaminate the hospital environment, form biofilms on medical devices, colonize the hands of healthcare workers, and contribute to healthcare-associated outbreaks (*14*,*21*). Outbreaks are more likely to occur when hospital services are strained, as occurred during the height of the pandemic.

Although *C. albicans* is a common colonizer of the skin and mucous membrane, a virulent species, and causes a high proportion of invasive infections, non–*C.* *albicans Candida* species have greater antifungal drug resistance (*22*,*23*). In South Africa, the species causing infection might be used as a proxy for azole resistance, particularly for *C. auris*, *N. glabratus*, *P. kudriavzevii*, and *C. parapsilosis*. For instance, *C. albicans* isolated from patients with BSI have the lowest incidence of azole resistance, whereas *N. glabratus* has decreased susceptibility across the azole class (*24*). On the basis of national laboratory-based *Candida* BSI surveillance data in South Africa from 2009–2010, almost 25% of isolates were resistant to fluconazole and 10% were resistant to voriconazole (*25*). At a tertiary care hospital in Johannesburg during 2016–2020, a total of 47% (201/426) of non–*C.* *albicans Candida* isolates were azole-resistant and 0.9% (4/491) were resistant to amphotericin B (*26*).

Overall, we demonstrated a change in the distribution of BSI *Candida* species isolated during 2019–2022; *C. auris* was the second most common species. An earlier national survey conducted during 2016–2017 found that *C. parapsilosis* was the most common species causing BSI and accounted for 44% (2,600/5,876) of cases, followed by *C. albicans* (23% [1,353/5,876]) and *C. auris* (14% [794/5,876]) (*27*).

Fifty percent of *Candida* BSI cases reported in our study were from the Gauteng Province, the most densely populated region of South Africa and the region with the most healthcare facilities and hospital beds (*25*,*28*). That province accounted for almost one third of *C. auris* cases. We found that *C. auris* caused more than one third of *Candida* BSI cases from the North West province. The Eastern Cape, KwaZulu-Natal, Limpopo, and Mpumalanga Provinces each accounted for >20% of *C. auris* cases in South Africa.

The observed changes in *C. auris* case numbers during the study period might have been caused by the patterns of COVID-19 through the pandemic waves (*29*). Alternatively, *C. auris* has spread out of Gauteng Province to cause outbreaks in multiple new facilities. Of concern, the incidence of *C. auris* in Northern Cape Province might be underestimated in the public sector. That finding might reflect differences in the extent of laboratory investigation of BSI between the public and private sectors in the Northern Cape Province.

The first limitation of our study is that overall, more than two thirds of all cases of *Candida* BSI were reported from the private sector in South Africa and half were reported from the Gauteng Province. Fewer cases of *Candida* BSI were reported from the public sector. Because we excluded *Candida* species other than the 6 most common species, we might have undercounted cases in the public sector because some laboratories either did not identify *Candida* to species level or misclassified the common *Candida* species as rarer species (*22*). Not all the data from the private laboratories were included in this study, and therefore these data do not necessarily represent the overall epidemiology of *Candida* BSI in the private sector. The prevalence and incidence of *Candida* BSI in South Africa could not be determined because denominator data were not available. Furthermore, we could not determine whether any cases were co-infections with *Candida* and SARS-CoV-2.

In conclusion, we identified changes in the distribution of *Candida* species in both the private and public heath sectors and in different provinces in South Africa during 2019–2021, particularly for *C. auris*. From 2017 to 2019, *C. auris* increased from 14% to 17%. However, large percentage increases occurred during the COVID-19 pandemic in 2020 (24%), 2021 (31%), and 2022 (28%). Taken together with temporal increases after each wave, our findings suggest that the COVID-19 pandemic might have driven the major increase in *C. auris* and thus accelerated the epidemiologic shift of *C. auris* in South Africa.

Appendix Additional information about accelerated increase in *Candida auris* bloodstream infections during COVID-19 pandemic, South Africa.
