# Supplementary material for: Accelerated Increase in Candida auris Bloodstream Infections during COVID-19 Pandemic, South Africa
Source: Emerg Infect Dis. 2026 Apr;32(4):563–72. doi: 10.3201/eid3204.251407 (PMC13094847; doi:10.3201/eid3204.251407)
Supplement: Appendix — Additional information about accelerated increase in Candida auris bloodstream infections during COVID-19 pandemic, South Africa. [file 25-1407-Techapp-s1.pdf]

Article DOI: https://doi.org/10.3201/eid3203.251407

EID cannot ensure accessibility for supplementary materials supplied by authors. Readers who have difficulty accessing supplementary content should contact the authors for assistance.

Accelerated Increase in *Candida auris* Bloodstream Infections during COVID-19 Pandemic, South Africa

Appendix

Appendix Table. Breakdown of *Candida* bloodstream infection by province in South Africa. January 2019 to June 2022 (N = 15393).

		2019		2020		2021		2022		Total		
Category	**Province**	**n**	**%**	**n**	**%**	**n**	**%**	**n**	**%**	**n**	**%**	**p-value**
**National**	Eastern Cape	277	8	315	7	371	7	196	8	1159	8	0.18
	Free State	207	6	143	3	144	3	104	4	598	4	<0.01
	Gauteng	1633	48	2078	49	2785	52	1210	50	7706	50	<0.01
	KwaZulu-Natal	507	15	619	15	707	13	330	14	2163	14	0.11
	Limpopo	115	3	143	3	123	2	81	3	462	3	<0.01
	Mpumalanga	99	3	157	4	223	4	108	4	587	4	0.01
	Northern Cape	14	0.4	19	0.4	46	1	25	1	104	1	<0.01
	North West	186	5	149	4	276	5	138	6	749	5	<0.01
	Western Cape	368	11	577	14	627	12	237	10	1809	12	<0.01
	Unknown	9	0.3	29	1	13	0.2	5	0.2	56	0.4	
	**Total**	**3415**	**100**	**4229**	**100**	**5315**	**100**	**2434**	**100**	**15393**	**100**	
**Private**	Eastern Cape	153	6	179	6	234	6	115	7	681	6	0.72
	Free State	130	5	82	3	70	2	51	3	333	3	<0.01
	Gauteng	1244	52	1419	49	2042	53	870	52	5575	51	0.07
	KwaZulu-Natal	341	14	434	15	531	14	238	14	1544	14	0.42
	Limpopo	70	3	70	2	60	2	49	3	249	2	<0.01
	Mpumalanga	66	3	111	4	153	4	60	4	390	4	0.08
	Northern Cape	12	1	18	1	46	1	25	1	101	1	<0.01
	North West	159	7	124	4	253	7	125	7	661	6	<0.01
	Western Cape	209	9	403	14	478	12	146	9	1236	11	<0.01
	Unknown	9	0.4	29	1	13	0.3	5	0.3	56	1	
	**Total**	**2393**	**100**	**2869**	**100**	**3880**	**100**	**1684**	**100**	**10826**	**100**	
**Public**	Eastern Cape	124	12	136	10	137	10	81	11	478	10	0.19
	Free State	77	8	61	4	74	5	53	7	265	6	<0.01
	Gauteng	389	38	659	48	743	52	340	45	2131	47	<0.01
	KwaZulu-Natal	166	16	185	14	176	12	92	12	619	14	0.02
	Limpopo	45	4	73	5	63	4	32	4	213	5	0.54
	Mpumalanga	33	3	46	3	70	5	48	6	197	4	<0.01
	Northern Cape	2	0.2	1	0.1	0	0	0	0	3	0.1	0.25
	North West	27	3	25	2	23	2	13	2	88	2	0.29
	Western Cape	159	16	174	13	149	10	91	12	573	13	<0.01
	Unknown	0	0	0	0	0	0	0	0	0	0	
	**Total**	**1022**	**100**	**1360**	**100**	**1435**	**100**	**750**	**100**	**4567**	**100**	

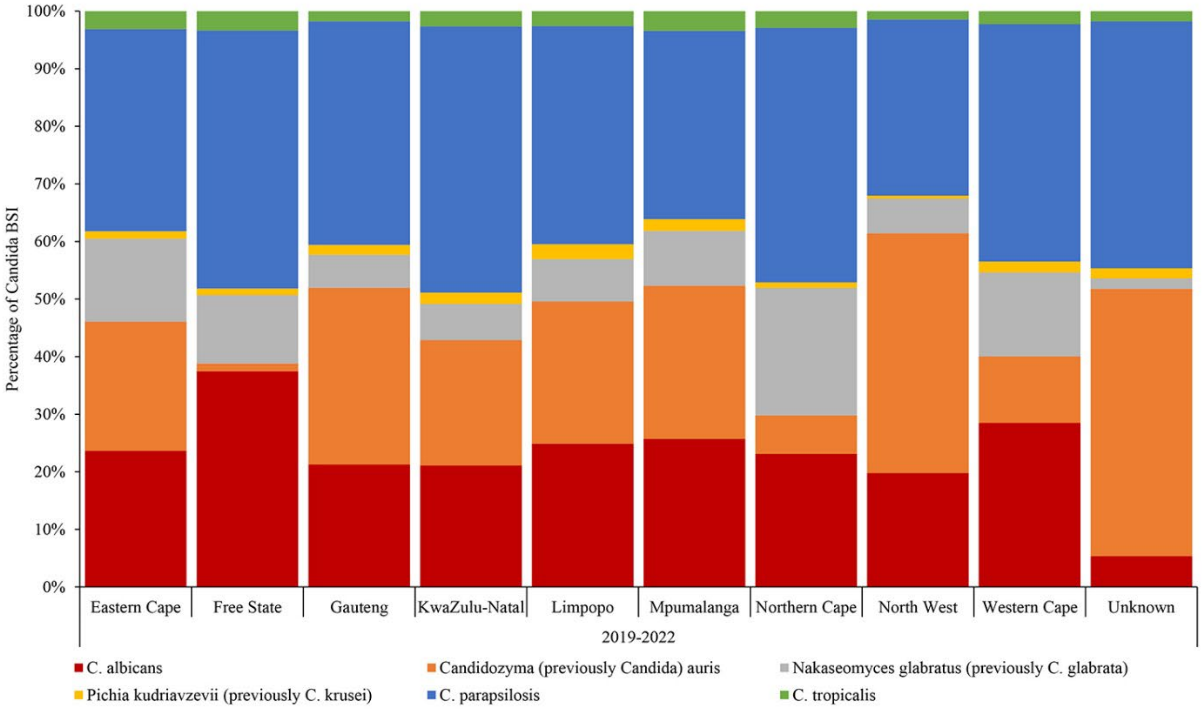

Appendix Figure. Percentage distribution of six *Candida* species by province in South Africa, January 2019 to June 2022 (N = 15393).
